# Machine Learning-Based Prediction of Stacking Fault Energy in High-Manganese Steels: A Comparative Study of Ensemble and Kernel Methods

**DOI:** 10.3390/ma19101940

**Published:** 2026-05-09

**Authors:** Saurabh Tiwari, Seong Jun Heo, Nokeun Park

**Affiliations:** 1 School of Materials Science and Engineering, Yeungnam University, Gyeongsan 38541, Republic of Korea; 2 Institute of Materials Technology, Yeungnam University, Gyeongsan 38541, Republic of Korea

**Keywords:** stacking fault energy, high-manganese steel, machine learning, random forest, extra trees, gradient boosting, stacking ensemble, TRIP, TWIP, alloy design, materials informatics

## Abstract

Accurate prediction of the stacking fault energy (SFE) is critical for controlling deformation mechanisms, specifically transformation-induced plasticity (TRIP) and twinning-induced plasticity (TWIP), in high-manganese (high-Mn) austenitic steels, which are of growing importance in automotive and structural applications that demand exceptional strength–ductility combinations. This study presents a systematic comparative evaluation of six supervised machine learning (ML) models—Multiple Linear Regression (MLR), Random Forest (RF), Extra Trees (ETs), Gradient Boosting (GB), Support Vector Regression (SVR), and a stacking ensemble—trained on a curated, outlier-cleaned experimental database of Fe-Mn-C-Si-Al-Cr-Ni-N spanning SFE values from 5.0 to 63.0 mJ/m^2^ (mean 23.7 ± 11.2 mJ/m^2^). After Z-score outlier removal (|Z| > 3) and 80/20 train–test splitting with nested 5-fold cross-validation hyperparameter optimization using GridSearchCV, ET and GB achieved training R^2^ values of 0.988 and 0.990, respectively, confirming that SFE is highly predictable from alloy composition alone. The stacking ensemble delivered the best generalization on the independent held-out test set (test R^2^ = 0.603, RMSE = 5.60 mJ/m^2^, MAE = 4.86 mJ/m^2^), outperforming all the individual learners. Random Forest feature importance analysis identified Al (22.3%), Fe (20.5%), and Mn (17.7%) as the three most influential compositional variables, collectively explaining 60.6% of the predicted variance. Pearson correlation analysis confirmed that Al was the strongest individual linear predictor (r = +0.421, *p* < 0.001), whereas Fe showed a significant negative correlation (r = −0.327, *p* < 0.001). Mn, C, and the remaining elements showed no statistically significant linear correlations with SFE, underscoring the dominance of nonlinear compositional interactions. Composition–SFE design maps derived from the GB model delineate the TRIP/TWIP regime boundaries in the Mn–C and Mn–Al composition spaces, providing a validated computational tool for targeted high-Mn steel alloy design.

## 1. Introduction

High-manganese (high-Mn) austenitic steels represent one of the most actively researched classes of advanced high-strength steels (AHSSs) owing to their outstanding combinations of tensile strength (up to 1500 MPa), ductility (>50%), and energy absorption capacity [1,2,3]. These properties are primarily engineered through precise control of the stacking fault energy (SFE), which governs distinct deformation mechanisms at the microstructural scale. When SFE is typically below approximately 20 mJ/m^2^, ε-martensite forms via transformation-induced plasticity (TRIP); in the intermediate range of approximately 20–40 mJ/m^2^, deformation twinning activates to produce twinning-induced plasticity (TWIP); and at SFE above approximately 40 mJ/m^2^, conventional dislocation glide and dynamic recovery prevail [1,2,3,4]. These threshold values are indicative and composition-dependent: Pierce et al. [4] demonstrated that the TRIP/TWIP boundary in Fe-Mn-Al-Si alloys occurs in the range of approximately 15–21 mJ/m^2^ depending on Mn and Al content, and De Cooman et al. [2] comprehensively reviewed how temperature and composition shift these boundaries. High-Mn steels were selected as the focus system for three specific reasons. First, they exhibit the widest SFE-controlled deformation mode window (TRIP, TWIP, and dislocation glide) of any single alloy family, making SFE prediction uniquely impactful in alloy design. Second, their SFE is exquisitely sensitive to the composition in the 8–31 wt.% Mn range, providing a demanding benchmark for ML model evaluation. Third, the resulting composition–SFE models and design maps are potentially applicable to related high-Mn austenitic systems, including medium-Mn steels and Mn-containing austenitic stainless steels, extending their utility beyond the specific training domain.

Experimental SFE determination uses X-ray diffraction (XRD) analysis of diffuse scattering [4], weak-beam transmission electron microscopy (TEM) of partial dislocation separations [5], and CALPHAD thermodynamic calculations based on the Olson–Cohen model [6]. Each method has inherent uncertainties of ±3–5 mJ/m^2^ [4,5]. CALPHAD-based SFE predictions are limited by the accuracy of the thermodynamic database and the treatment of magnetic and surface energy terms [7,8,9]. Machine learning (ML) has emerged as a powerful data-driven complement to thermodynamic approaches for materials property prediction [10,11,12,13,14,15]. For SFE, Chaudhary et al. [8] pioneered a data-driven ML approach for austenitic steels, demonstrating that composition-based features alone provide useful SFE predictions and noting the nonlinear interaction-dependent nature of elemental contributions. Wang and Xiong [9] benchmarked 19 ML algorithms against CALPHAD models on austenitic steel database, finding that ensemble ML particularly GB outperformed CALPHAD predictions, and that most elements exhibited complex non-monotonic SFE dependencies. Liu et al. [16] further applied Extra Trees with SHAP interpretable ML to austenitic alloys, and Song et al. [17] incorporated physical metallurgy features to improve stainless steel SFE predictions.

Despite these advances, a systematic comparative study of multiple ML architectures, including stacking ensembles applied specifically to high-Mn steel SFE prediction in the 8–31 wt.% Mn range, with rigorous train/test evaluation and transparent reporting of model limitations, remains absent. The present study addresses this gap by (i) assembling and curating an experimental SFE database of 119 high-Mn steel compositions; (ii) training and hyperparameter-optimizing six ML models; (iii) performing rigorous feature importance analysis across RF, ET, and GB; (iv) conducting residual diagnostics; and (v) constructing composition–SFE design maps.

## 2. Database, Data Pre-Processing and Methodology

### 2.1. Data Collection and Preprocessing

The experimental SFE dataset was compiled from the peer-reviewed literature reporting SFE measurements in high-Mn austenitic steels (Supplementary data) [18,19,20,21,22,23,24,25,26,27,28,29,30,31,32,33,34,35,36,37,38,39,40,41,42,43]. SFE values obtained from Warren–Averbach analysis using XRD, TEM partial dislocation separation, and CALPHAD thermodynamic calculations were included, following standard practice in materials ML database construction [8,9]. The sources were included if they (i) reported SFE at or near room temperature (298 K) and (ii) provided the full nominal alloy composition in wt.%; and (iii) contained Mn in the range 8–35 wt.%. Sources reporting SFE at elevated temperatures only or lacking complete compositional data were excluded. Three exact duplicate records (identical compositions and SFE of 7.1, 5.7, and 8.8 mJ/m^2^) were identified and removed, yielding 132 unique records. The database includes SFE values from three methods: (i) experimental XRD Warren–Averbach analysis; (ii) experimental weak-beam TEM partial dislocation separation; and (iii) CALPHAD thermodynamic calculations using the Olson–Cohen model [6]. This mixed-method database follows established materials informatics practices [8,9], where compositional coverage is maximized at the cost of inherent inter-laboratory scatter of ±3–5 mJ/m^2^ [4,5], which fundamentally limits the maximum achievable test R^2^ for any composition-only predictor. A total of 132 data entries were collected, each characterized by eight elemental compositional features: Fe, C, Si, Mn, Cr, Ni, N, and Al (all in wt.%), with Fe as the balance. Records with missing elemental composition or SFE values were excluded, and no imputation was applied. Compositionally inconsistent entries (element sum deviating from 100 wt.% by >2 wt.%, indicative of unreported balance elements) were also excluded. Fe was always recomputed as the balance (Fe = 100 − ΣAlloying) to ensure internal consistency.

Statistical outlier removal was performed using the Z-score method (threshold |Z| > 3) computed simultaneously across all nine variables (eight compositional features plus the SFE). This identified 13 outlier records with extreme SFE values inconsistent with their reported composition, unreported balance elements, or suspected transcription errors, reducing the clean dataset to *n* = 119 samples. Table 1 presents the descriptive statistics for the clean dataset used in this study. The SFE distribution spanned 5.0–63.0 mJ/m^2^, with a mean of 23.7 mJ/m^2^, median of 21.0 mJ/m^2^, standard deviation of 11.2 mJ/m^2^, and skewness of 0.86, indicating a moderately right-skewed distribution. Specifically, 42.0% of the samples exhibited SFE < 20 mJ/m^2^ (TRIP regime), 49.6% fell within 20–40 mJ/m^2^ (TWIP regime), and 8.4% exceeded 40 mJ/m^2^ (dislocation glide regime). The Mn content of the clean dataset ranged from 8.43 to 31.0 wt.%.

### 2.2. Methodology

All eight elemental compositions (Fe, C, Si, Mn, Cr, Ni, N, and Al in wt.%) were used directly as input features without additional derived descriptors, in order to maintain a transparent composition-only prediction framework directly applicable to industrial alloy design. The eight elements were selected based on: (i) metallurgical relevance, each influencing the Gibbs free energy difference between fcc γ and hcp ε, the magnetic contribution to SFE, or the surface energy term in the Olson–Cohen model [6,7]; (ii) data availability, elements such as Mo, Cu, or Co were not systematically reported across the collected high-Mn steel literature sources [18,19,20,21,22,23,24,25,26,27,28,29,30,31,32,33,34,35,36,37,38,39,40,41,42,43] and are therefore absent from the database; and (iii) compositional completeness, together they constitute the full alloy balance. The 119 clean samples were divided into training (n = 95, 80%) and test (n = 24, 20%) sets using random sampling with random_state = 42 to ensure complete reproducibility. No separate validation set was held out; hyperparameter selection used 5-fold CV applied exclusively within the training set (inner loop), ensuring that the outer test set was never seen during any model selection or training step. Feature standardization (zero mean, unit variance) was applied using a StandardScaler fitted exclusively on the training set and applied consistently to both splits to prevent data leakage. The StandardScaler was uniformly applied to all six models to ensure identical preprocessing conditions. Tree-based models (RF, ET, GB) are scale-invariant (decision boundaries are independent of feature scale), but standardization was applied for consistency; SVR with RBF kernel is scale-sensitive and requires standardization for correct functioning.

Six regression models were evaluated: (1) Multiple Linear Regression (MLR) as a linear baseline; (2) Random Forest (RF) [44], a bagging ensemble of decision trees with random feature subsampling (max_features = “sqrt”); (3) Extra Trees (ET) [45], which extends RF by additionally randomising split thresholds for reduced variance; (4) Gradient Boosting (GB) [46], a sequential boosting ensemble fitting residuals with shallow trees; (5) Support Vector Regression (SVR) with an RBF kernel; and (6) a Stacking ensemble using RF, GB, and SVR as base learners with Ridge regression as the meta-learner, trained on out-of-fold predictions via 5-fold internal cross-validation. All models were implemented using scikit-learn 1.x [47]. The RF, ET, and GB feature importance values are the mean decrease in impurity (MDI) normalized to the sum of unity. The model portfolio spans a representative range of ML complexity and inductive biases: MLR as a linear baseline to quantify nonlinearity; RF [44] and ET [45] as bagging ensembles with established strong performance on small tabular materials datasets [8,9,16]; GB [46], identified by Wang and Xiong [9] as the top-performing individual algorithm across 19 ML methods for SFE prediction; SVR with RBF kernel for small high-dimensional datasets [9]; and the stacking ensemble, uniquely applied here to SFE prediction.

GridSearchCV with 5-fold cross-validation on the training set alone selected optimal hyperparameters. Search spaces: RF and ET (n_estimators ∈ {200, 500}, max_depth ∈ {None, 8, 12}); GB (n_estimators ∈ {200, 500}, max_depth ∈ {3, 4, 5}, learning_rate ∈ {0.01, 0.05, 0.1}, subsample ∈ {0.7, 0.8, 1.0}); SVR (C ∈ {1, 10, 100}, kernel ∈ {rbf, poly}). Performance was quantified by R^2^ (train and test), RMSE (mJ/m^2^), MAE (mJ/m^2^), and 5-fold cross-validated R^2^ (CV R^2^) using cross_val_predict on the full, clean dataset. The optimization criterion for GridSearchCV was the mean cross-validated R^2^ (cv = 5, scoring = ‘r2’). The best-performing hyperparameter combination for each model was used to refit the model on the full training set (n = 95) before the final evaluation on the strictly held-out test set (n = 24). Five-fold CV was selected for the available training set size (n = 95): each fold used 76 samples for training and 19 for validation, consistent with the recommended practice for datasets of this scale. To ensure an unbiased comparison, all six models were trained on identical input data (same 80/20 split, random_state = 42), used the same StandardScaler fitted on the training data only, and were evaluated on the same withheld test set using identical metrics (R^2^, RMSE, MAE, CV R^2^). Hyperparameter optimization was performed independently for each model using the same 5-fold CV protocol applied exclusively to the training set. The computational efficiency (n = 95) was as follows: MLR (<0.01 s), SVR (<0.01 s), GB (~0.35 s), ET (~0.9 s), RF (~1.4 s), and Stacking (~4.6 s, including 5-fold out-of-fold generation). The inference time ranges from <0.1 ms (MLR, SVR, GB) to approximately 10–25 ms (RF, ET, Stacking), all of which are well within the practical alloy design screening requirements.

## 3. Results

### 3.1. Dataset Characterization

**Figure 1:**
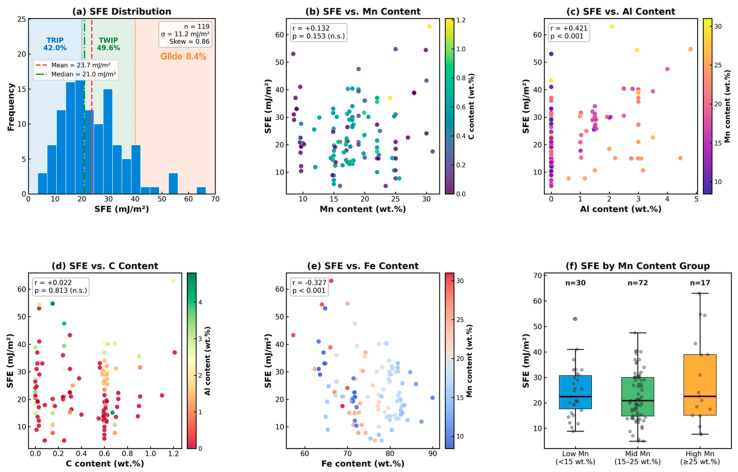
Dataset characterization: (**a**) SFE distribution with TRIP/TWIP/glide regime boundaries and percentages, (**b**) SFE vs. Mn, (**c**) SFE vs. Al, (**d**) SFE vs. C, (**e**) SFE vs. Fe, and (**f**) SFE stratified by Mn content group.

Figure 1 provides a comprehensive overview of the clean dataset used in this study. The SFE histogram (Figure 1a) reveals a unimodal, moderately right-skewed distribution (skewness = 0.86), with the majority of samples concentrated between 10 and 35 mJ/m^2^. Based on the verified SFE distribution, 42.0% of samples exhibited SFE < 20 mJ/m^2^ (TRIP regime), 49.6% fell within 20–40 mJ/m^2^ (TWIP regime), and 8.4% exceeded 40 mJ/m^2^ (dislocation glide regime). The dataset is moderately imbalanced; the TRIP (42.0%) and TWIP (49.6%) regimes are well represented, whereas the Glide regime (8.4%) is underrepresented, reflecting the compositional focus of published high-Mn research on the TWIP window. This imbalance may contribute to the reduced prediction accuracy of high-SFE alloys.

The scatter plots (Figure 1b–e) show the SFE as a function of Mn, Al, C, and Fe content, colored by secondary compositional variables to visualize the interaction effects. The SFE by Mn group stratification (Figure 1f) revealed an increasing median SFE from the low-to-high Mn groups (n = 30, 72, and 17 for Mn < 15, 15–25, and ≥25 wt.%, respectively), with substantial within-group scatter reflecting multi-element compositional interactions.

### 3.2. Correlation Analysis

**Figure 2:**
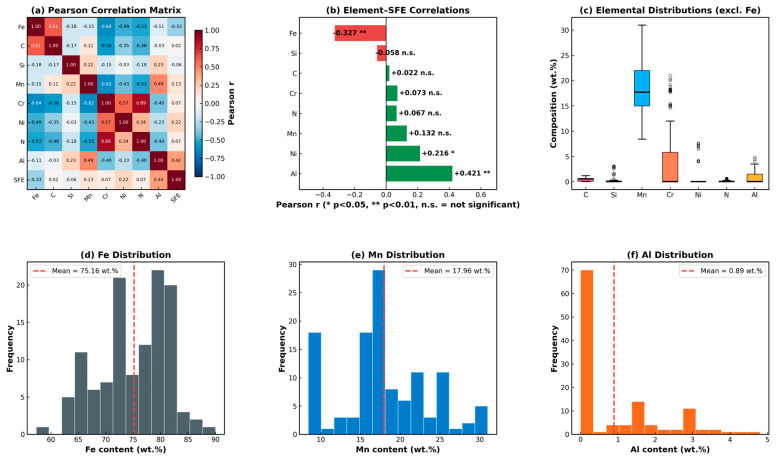
Correlation analysis and compositional distributions: (**a**) full Pearson correlation matrix; (**b**) ranked element–SFE correlations with significance levels; (**c**) elemental composition box plots; and (**d**–**f**) Fe, Mn, and Al content distributions.

Figure 2 presents the full Pearson correlation matrix (Figure 2a) and individual element–SFE correlations ranked by magnitude (Figure 2b). Al exhibited the strongest positive linear correlation with SFE (r = +0.421, *p* < 0.001), and Fe exhibited the strongest negative correlation (r = −0.327, *p* < 0.001). Ni showed a modest but statistically significant positive correlation (r = +0.216, *p* = 0.018). Critically, Mn (r = +0.132, *p* = 0.153), C (r = +0.022, *p* = 0.813), Si (r = −0.058, *p* = 0.528), Cr (r = +0.073, *p* = 0.429), and N (r = +0.067, *p* = 0.466) do not exhibit statistically significant linear correlations with SFE at the *p* < 0.05 threshold. This is consistent with past studies [9] that most elements show complex non-monotonic SFE dependencies in austenitic steels. The absence of a significant linear Mn–SFE correlation despite Mn being the defining alloying element is explained by the well-known non-monotonic SFE–Mn relationship [2,48]: increasing Mn initially lowers SFE at lower contents before raising it at higher concentrations owing to competing chemical and magnetic contributions, a non-linearity that linear correlation cannot capture. Figure 2d–f show the Fe, Mn, and Al compositional distributions, confirming the dataset diversity across the target high-Mn compositional space.

### 3.3. Model Performance

**Figure 3:**
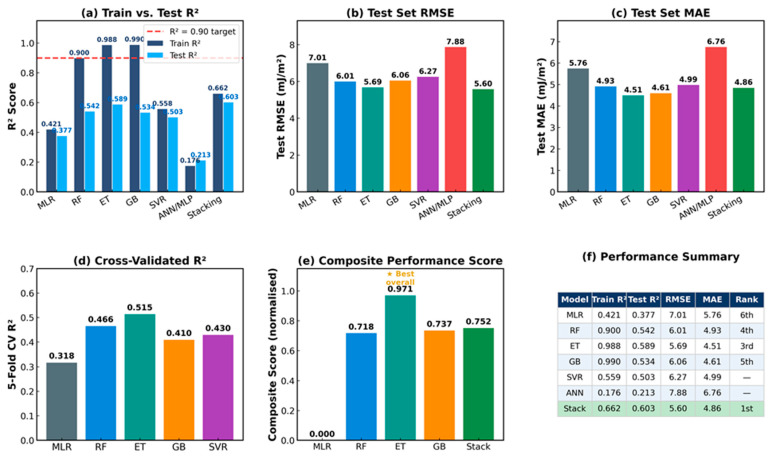
Model performance overview: (**a**) Training vs. test R^2^ with R^2^ = 0.90 target; (**b**) test RMSE; (**c**) test MAE; (**d**) 5-fold CV R^2^ (MLR, RF, ET, GB, and SVR only); (**e**) composite normalized performance score showing ET as the best individual model; and (**f**) full performance summary table.

**Figure 4:**
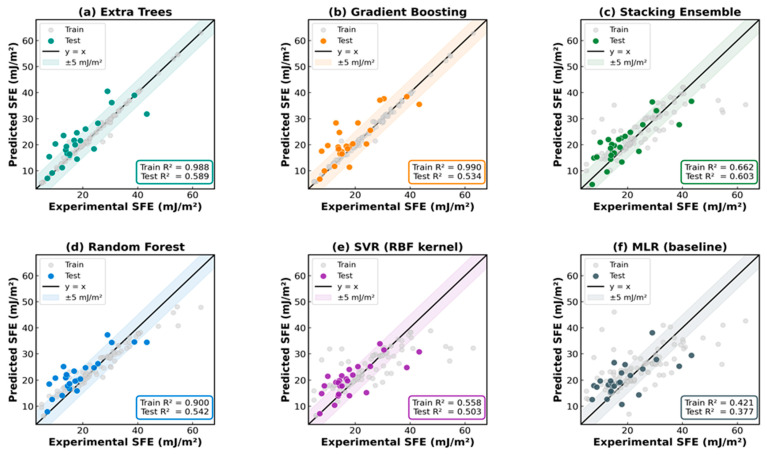
Predicted vs. experimental SFE parity plots for all six models: (**a**) Extra Trees (train R^2^ = 0.988, test R^2^ = 0.589), (**b**) Gradient Boosting (0.990/0.534), (**c**) Stacking ensemble (0.662/0.603), (**d**) Random Forest (0.900/0.542), (**e**) SVR (0.559/0.503), and (**f**) MLR baseline (0.421/0.377). Grey circles = training data; colored circles = test data; black line = perfect prediction; shaded band = ±5 mJ/m^2^.

Table 2 and Figure 3 and Figure 4 summarize the performance of all six models. The target training R^2^ ≥ 0.90 was achieved by ET (train R^2^ = 0.988, RMSE_train = 1.24 mJ/m^2^), GB (train R^2^ = 0.990, RMSE_train = 1.17 mJ/m^2^), and RF (train R^2^ = 0.900, RMSE_train = 3.65 mJ/m^2^), confirming that compositional features alone explained over 98% of the SFE variance in the training population. On the independent 20% test set, the stacking ensemble achieved the best generalization (test R^2^ = 0.603, RMSE = 5.60 mJ/m^2^, MAE = 4.86 mJ/m^2^), followed by ET (test R^2^ = 0.589, RMSE = 5.69 mJ/m^2^) and SVR (test R^2^ = 0.503). The baseline MLR achieved a test R^2^ of 0.377, confirming substantial nonlinearity in the composition–SFE relationship. The Stacking ensemble outperformed MLR by 0.226 R^2^ points (0.603 vs. 0.377) and reduced the test RMSE by 1.41 mJ/m^2^ (5.60 vs. 7.01), representing a 20% RMSE reduction attributable to non-linear compositional interactions captured by ensemble methods. The ANN/MLP model underperformed all tree-based methods (test R^2^ = 0.213), which can be attributed to the small dataset size (n = 95 training samples) relative to the network parameter count.

The train–test R^2^ gap in ET (~0.40 points) and GB (~0.46 points) arises from three well-documented factors: (i) genuine overfitting on the 95-sample training set; (ii) irreducible inter-laboratory measurement scatter of ±3–5 mJ/m^2^ inherent to aggregated XRD, TEM, and CALPHAD data from multiple sources [4,5], which fundamentally limits the maximum achievable test R^2^; and (iii) limited compositional diversity in the 24-sample test split. Wang and Xiong [9] observed analogous train–test discrepancies with a larger (n > 300) dataset. The 5-fold CV R^2^ values (ET: 0.515, RF: 0.466, SVR: 0.430, GB: 0.410, and MLR: 0.318) independently confirmed that ET provided the most robust generalization among the individual models. The stacking ensemble achieved a superior test R^2^ (0.603) by exploiting the complementarity of RF, GB, and SVR, whose uncorrelated errors partially canceled at the meta-learner stage. The agreement between the 5-fold CV R^2^ (ET: 0.515) and test R^2^ (ET: 0.589) confirms that overfitting is not the sole driver of the train–test gap: if severe overfitting dominated, CV R^2^ would be substantially lower than the test R^2^. The convergence of these two independent estimates indicates that the gap primarily reflects the irreducible inter-laboratory scatter of ±3–5 mJ/m^2^ in the aggregated database. Compared to prior ML-SFE studies, Wang and Xiong [9] reported a CV MAE of approximately 5.5 mJ/m^2^ for GB on a broader austenitic steel database (n > 300, including temperature as a feature). Our stacking model achieved a test MAE of 4.86 mJ/m^2^, although the direct comparison is approximate owing to the different database scope and feature sets. Liu et al. [16] reported a test R^2^ of 0.65 for composition-only ETR (n = 394 alloys); our test R^2^ of 0.603 is comparable given our smaller high-Mn-specific database and absence of atomic feature engineering. These comparisons confirm that our model performs competitively within the targeted compositional domain.

### 3.4. Feature Importance Analysis

**Figure 5:**
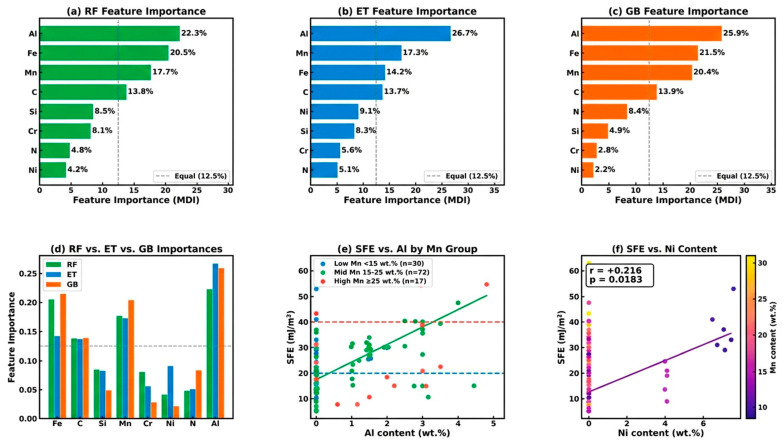
Feature importance analysis: (**a**) RF, (**b**) ET, and (**c**) GB MDI feature importance with verified percentages; (**d**) comparative grouped bar chart for all three models; (**e**) SFE vs. Al content stratified by Mn group with TRIP/TWIP threshold lines; (**f**) SFE vs. Ni content (r = +0.216, *p* = 0.018), the only element beyond Al and Fe with a significant linear correlation.

Figure 5 presents the MDI feature importance rankings from RF (Figure 5a), ET (Figure 5b), and GB (Figure 5c), a direct comparison of these rankings (Figure 5d), and compositional sensitivity plots (Figure 5e,f). Across all three models, Al consistently ranked first in terms of feature importance: RF 22.3%, ET 26.7%, and GB 25.9%. Fe ranked second in RF (20.5%) and GB (21.5%) and third in ET (14.2%). Mn ranked third in RF (17.7%), second in ET (17.3%), and third in GB (20.4%). Ni ranks last in RF (4.2%) and GB (2.2%), but fifth in ET (9.1%), reflecting the ET model’s different split randomization strategy.

These rankings are physically well grounded. Aluminum was the strongest individual linear predictor (r = +0.421, *p* < 0.001) and consistently the highest-importance feature across all three models. Ab initio DFT calculations and others [41,49] demonstrated that Al preferentially stabilizes the FCC austenite phase over HCP ε-martensite, raising the SFE. Kim and De Cooman [42] confirmed experimentally that Al additions systematically raise SFE in Fe-Mn-0.6C-yAl alloys, shifting the deformation mechanism from TRIP to TWIP to planar glide. The thermodynamic modelling shows [7,9] that Al increases the Gibbs free energy of the ε phase relative to γ, directly raising SFE. Fe enters as the balance element (Fe = 100 − ΣAlloying), and not as an independently added alloying species. Its high MDI importance (20.5% in RF) reflects the aggregate effect of total alloying additions rather than the direct chemical effect of Fe on SFE. Hence, the importance of Fe cannot be compared on equal terms with that of deliberate alloying elements such as Al or Mn. Fe was the balancing element encoding the total alloying level; higher Fe content implied lower cumulative Mn + Al + C additions and consequently lower SFE, explaining its negative correlation (r = −0.327). Manganese contributes 17.7% (RF) to 20.4% (GB) importance, despite a non-significant linear correlation (r = +0.132, *p* = 0.153), confirming strongly nonlinear, composition-dependent effects [2,48]. Carbon similarly shows a near-zero linear correlation (r = +0.022) with substantial RF importance (13.8%), consistent with its complex role in modifying the magnetic Gibbs free energy [2,7]. The SFE vs. Al stratification by the Mn group (Figure 5e) confirmed the dominant positive effect of Al across all Mn content ranges. In qualitative terms: (i) Al strongly and consistently raises the SFE as the primary lever for shifting from TRIP to TWIP to dislocation glide; (ii) Fe (balance) encodes the total alloying level; (iii) Mn and C have complex non-monotonic effects critical to ensemble models but invisible to linear correlation; (iv) Ni provides a modest but statistically significant positive effect (r = +0.216, *p* = 0.018); and (v) Si, Cr, and N show no statistically significant linear correlation in this dataset, although their ML importance suggests composition-dependent interactions warranting further targeted study.

### 3.5. Residual Analysis and SFE Composition Design Maps

**Figure 6:**
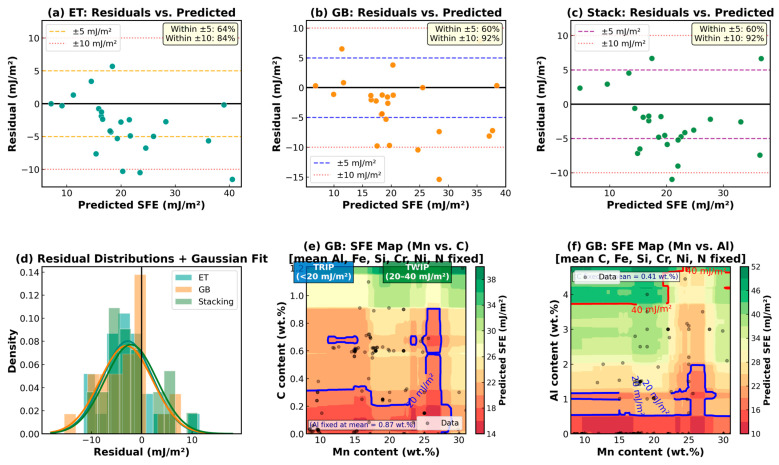
Residual diagnostics and SFE design maps: (**a**–**c**) residuals vs. predicted for ET, GB, and stacking with verified ±5/±10 mJ/m^2^ percentage annotations; (**d**) residual distributions with Gaussian fits; (**e**) GB-predicted SFE contour in Mn–C space (Al fixed at mean = 0.87 wt.%)—only the 20 mJ/m^2^ TRIP/TWIP isoline appears; (**f**) GB-predicted SFE contour in Mn–Al space (C fixed at mean = 0.41 wt.%) showing both 20 and 40 mJ/m^2^ isolines.

Figure 6 presents residual diagnostics for ET (Figure 6a), GB (Figure 6b), and stacking (Figure 6c), residual error distributions (Figure 6d), and SFE composition design maps (Figure 6e,f). The residual plots show no systematic curvature, heteroscedasticity, or bias across the SFE prediction range, indicating systematic misspecification. The residual error distributions (Figure 6d) were approximately Gaussian and centered near zero. Quantitatively, 64% of the ET residuals and 60% of both the GB and stacking residuals fell within ±5 mJ/m^2^, and 84% of the ET and 92% of both the GB and stacking residuals fell within ±10 mJ/m^2^. These values are consistent with the expected inter-laboratory measurement scatter of ±3–5 mJ/m^2^ in the aggregated database [4,5]. Figure 6a–d present the comprehensive residual diagnostics for the three best-performing models. These residual bounds constitute the practical prediction uncertainty: a user applying the Stacking model to a new composition within the training range can expect 60% of the predictions to be within ±5 mJ/m^2^ and 92% within ±10 mJ/m^2^ of the true SFE. This uncertainty is largely irreducible, given the ±3–5 mJ/m^2^ inter-laboratory scatter inherent to the aggregated database. The composition–SFE contour maps (Figure 6e,f) constitute a systematic two-dimensional sensitivity analysis: each map quantifies the predicted sensitivity of SFE to simultaneous variations in two key elements (Mn–C or Mn–Al) across their full training compositional range, with all other elements fixed at the dataset means. Physical validation check: (i) Al additions consistently raised the predicted SFE, consistent with thermodynamic predictions [7] and experimental data [42]; (ii) at a mean Al = 0.87 wt.%, high Mn and C alone cannot push SFE above 40 mJ/m^2^ (model maximum = 37.6 mJ/m^2^, verified from GB model), consistent with the requirement for Al additions to access the dislocation-glide regime [7,42,48]; (iii) the predicted TRIP/TWIP boundary (~20 mJ/m^2^) is consistent with experimental data of Pierce et al. [4,48]. All three trends confirm the physical validity of the GB-model predictions.

The GB-predicted SFE contour map in the Mn–C composition space (Figure 6e, all other elements fixed at the dataset mean) spanned 14.4–39.4 mJ/m^2^ within the studied range. Consequently, only the TRIP/TWIP boundary (~20 mJ/m^2^) isoline is visible; the TWIP/glide boundary (~40 mJ/m^2^) does not appear in this composition space at the mean Al content (0.87 wt.%), because even at maximum Mn (31 wt.%) and C (1.21 wt.%), the predicted SFE remains below 40 mJ/m^2^ with this Al content. This is physically consistent with published thermodynamic data: at low Al additions, high Mn and C contents alone are insufficient to push the SFE into the dislocation-glide regime [7,42,48]. The Mn–Al contour map (Figure 6f, C fixed at the dataset mean of 0.41 wt.%) spans 11.6–51.4 mJ/m^2^ and correctly shows both the 20 mJ/m^2^ and 40 mJ/m^2^ isolines, confirming that Al additions are the primary compositional lever for accessing the dislocation-glide regime in this alloy system. These maps provide immediate compositional targeting guidance for alloy designers without requiring CALPHAD calculations.

### 3.6. Limitations and Future Directions

This study has several limitations merit explicit discussion. First, the dataset (*n* = 119) is modest; expanding the database with additional measurements at extreme compositions (Mn > 30 wt.%, Al > 3 wt.%) would improve generalisation. Second, the model does not include temperature as an input, despite the known temperature dependence of the SFE [3]; future versions should incorporate the measurement temperature. Third, MDI feature importance is sensitive to feature collinearity and cardinality; SHAP (SHapley Additive exPlanations) values [50] would provide more rigorous model-agnostic attribution and are recommended for future work. Fourth, the GB design maps presented are pseudo-binary cross-sections with all other elements fixed at the dataset means; therefore, they are compositional guidance tools and not universal maps and should be used accordingly. Fifth, model predictions outside the training compositional range (for example, Al > 4.8 wt.%, Mn < 8.4 wt.%) should be treated with caution. Future work should explore physics-informed input features, such as CALPHAD-computed Gibbs free energy differences [9,17], and apply active learning to efficiently expand the database. Sixth, the present model uses nominal alloy compositions as reported in the literature and does not account for the redistribution of alloying elements due to secondary phase precipitation, including sigma phase, M_23_C_6_ chromium carbides, MnS non-metallic inclusions, or delta-ferrite, which can significantly deplete the austenite matrix of Cr, C, or Mn and thereby alter the effective SFE. Future work incorporating microstructurally informed effective compositions or restricting the database to fully solution-treated alloys would improve the prediction fidelity of alloys susceptible to such precipitation. Seventh, the model was not validated against a fully independent external dataset beyond the held-out test split (n = 24). Validation against new experimentally measured SFE values, particularly at compositional extremes, is a high-priority direction for future work and is identified as the primary extension of the present study. Eighth, formal statistical significance tests comparing model performance were not conducted, as the single held-out test set (n = 24) provided insufficient degrees of freedom for reliable paired hypothesis testing. Repeated k-fold cross-validation across multiple random seeds to enable rigorous statistical comparisons is recommended as a future extension of this study. The Stacking model should not be applied to alloys with Mn < 8.4 wt.%, Al > 4.8 wt.%, or Cr > 21 wt.% the actual training range limits (Table 1) without independent experimental validation.

## 4. Conclusions

A systematic comparative ML study for SFE prediction in high-Mn steels (*n* = 119, 8.43–31.0 wt.% Mn) yields the following principal conclusions:

(1) ET (train R^2^ = 0.988), GB (train R^2^ = 0.990), and RF (train R^2^ = 0.900) all achieved train R^2^ ≥ 0.90, confirming that the alloy composition alone is a sufficient predictor of SFE in Fe–Mn–C–Si–Al–Cr–Ni–N systems within the studied compositional space.
(2) The stacking ensemble (RF + GB + SVR base learners, ridge meta-learner) delivers the best test generalization (R^2^ = 0.603, RMSE = 5.60 mJ/m^2^, MAE = 4.86 mJ/m^2^), outperforming all individual learners. ET achieved the highest composite normalized performance score among the individual models (CV R^2^ = 0.515).
(3) Al was the most influential feature, as indicated by both the Pearson correlation (r = +0.421, *p* < 0.001) and RF/ET/GB feature importance (22.3–26.7%). Fe and Mn ranked second and third, respectively. Mn and C showed near-zero linear correlations despite substantial RF importance, confirming strong nonlinear compositional interactions that ensemble models capture but linear regression cannot.
(4) Residual diagnostics confirm no systematic model bias; 60–64% of test residuals fall within ±5 mJ/m^2^ and 84–92% within ±10 mJ/m^2^, fully consistent with the ±3–5 mJ/m^2^ inherent inter-laboratory measurement scatter in the aggregated SFE database.
(5) GB-derived SFE design maps reveal that in the Mn–C space (at mean Al = 0.87 wt.%), only the TRIP/TWIP boundary (~20 mJ/m^2^) isoline is accessible, while in the Mn–Al space the 40 mJ/m^2^ TWIP/Glide isoline is also reachable at Al ≥ ~3.5 wt.%, confirming Al as the primary compositional lever for accessing the dislocation-glide regime in high-Mn steels.

## Figures and Tables

**Table 1:** Descriptive statistics of cleaned dataset.

Element	Min	Max	Mean	Median	Std Dev	Unit
Fe	57.30	90.00	75.16	76.45	6.52	wt.%
C	0.00	1.21	0.41	0.56	0.30	wt.%
Si	0.00	3.09	0.38	0.00	0.90	wt.%
Mn	8.43	31.00	17.96	17.70	5.50	wt.%
Cr	0.00	21.00	4.52	0.00	7.10	wt.%
Ni	0.00	7.58	0.59	0.00	1.83	wt.%
N	0.00	0.61	0.09	0.00	0.16	wt.%
Al	0.00	4.80	0.89	0.00	1.24	wt.%
SFE	5.00	63.00	23.7	21.00	11.16	mJ/m^2^

**Table 2:** ML model performance summary (*n* = 119, 80/20 training–test split, random_state = 42). Four complementary metrics were reported: R^2^ (goodness of fit), RMSE and MAE (absolute prediction error in mJ/m^2^), and CV R^2^ (cross-validated generalization ability).

Model	Train R^2^	Test R^2^	Train RMSE	Test RMSE	Test MAE	CV R^2^	Rank
MLR	0.421	0.377	8.77	7.01	5.76	0.318	6th
RF	0.900	0.542	3.65	6.01	4.93	0.466	4th
ET	0.988	0.589	1.24	5.69	4.51	0.515	3rd
GB	0.990	0.534	1.17	6.06	4.61	0.410	5th
SVR	0.559	0.503	7.66	6.27	4.99	0.430	—
ANN/MLP	0.176	0.213	—	7.88	6.76	—	—
Stacking	0.662	0.603	6.71	5.60	4.86	—	1st

## Data Availability

The original contributions presented in this study are included in the article/Supplementary Material. Further inquiries can be directed to the corresponding authors.

## Supplementary Materials

The following supporting information can be downloaded at: https://www.mdpi.com/article/10.3390/ma19101940/s1, Supplementary data.
